# Beyond preferential attachment: falling of stars and survival of superstars

**DOI:** 10.1098/rsos.220899

**Published:** 2022-08-24

**Authors:** Shahar Somin, Yaniv Altshuler, Alex ‘Sandy’ Pentland, Erez Shmueli

**Affiliations:** ^1^ Industrial Engineering Department, Tel Aviv University, Tel Aviv, Israel; ^2^ MIT, Media Lab, Cambridge, MA, USA

**Keywords:** network theory, network dynamics, node ranking, popularity dynamics

## Abstract

Numerous studies over the past decades established that real-world networks typically follow preferential attachment and detachment principles. Subsequently, this implies that degree fluctuations monotonically increase while rising up the ‘degree ladder’, causing high-degree nodes to be prone for attachment of new edges and for detachment of existing ones. Despite the extensive study of node degrees (absolute popularity), many domains consider node ranks (relative popularity) as of greater importance. This raises intriguing questions—what dynamics are expected to emerge when observing the ranking of network nodes over time? Does the ranking of nodes present similar monotonous patterns to the dynamics of their corresponding degrees? In this paper, we show that surprisingly the answer is not straightforward. By performing both theoretical and empirical analyses, we demonstrate that preferential principles do not apply to the temporal changes in node ranking. We show that the ranking dynamics follows a non-monotonous curve, suggesting an inherent partition of the nodes into qualitatively distinct stability categories. These findings provide plausible explanations to observed yet hitherto unexplained phenomena, such as how superstars fortify their ranks despite massive fluctuations in their degrees, and how stars are more prone to rank instability.

## 1. Introduction

During the last decades, significant research efforts have been directed towards enhancing the understanding of complex systems and the dynamics they undergo. Discovering the existence of seemingly ubiquitous meta-structures has enabled advancements in countless fields, employing a network-oriented approach. Specifically, structural patterns such as power-law degree distributions [1–3], temporal burstiness [4] and clustering sizes distributions [5] have been of particular importance for many domains, and provided the ability to model, analyse and predict the macroscopic and mesoscopic behaviour of many of today’s ‘real-world’ systems.

Beyond static structural patterns, vast research has been directed towards examining the evolution and dynamics of node degrees. Specifically, much has been discussed regarding the evident patterns of preferential attachment [6] and detachment [7] of nodes, demonstrating that the rate at which nodes gain or loose edges is a monotonically increasing function of their previous degrees. Although, to date, most studies have concentrated on analysing degree-based *absolute popularity* of nodes, a multitude of domains assign greater importance to node *relative popularity* exhibited by their ranked degrees.

Indeed, from science [8,9] to pop music, through economy [10,11], industry^1^ and online search engines [12], ranking items is in the heart of all social activities.

While node ranking has attracted the attention of the scientific community (e.g. [13–16]), a modelling of the entire system’s ranking evolution is still lacking. In particular, the degree-based preferential attachment and detachment hypotheses raise some fundamental questions that have yet to be addressed. First, do preferential attachment^2^ dynamics apply also to the ranking of nodes? That is, is the rate at which nodes gain or lose their relative popularity a monotonic function of their prior ranks? Second, if preferential patterns do not apply to ranking evolution, what is its functional form and which forces govern its demeanour?

In this paper, we attend to the above-stated questions by performing both theoretical and empirical analyses. We show for the first time that preferential principles do not apply to relative popularity evolution. In particular, we demonstrate that the functional form of the relative popularity evolution follows a non-monotonous, inverse U-shaped, curve. This functional form suggests the existence of a structural partition of the node set into four qualitatively distinct categories, each with different stability characteristics.

Our findings manifest a clear distinction between the evolution of the absolute and relative popularity measures. The exhibited non-monotonic evolution of node ranking alongside the difference between nodes' stability levels, may shed a new light on observed yet hitherto unexplained phenomena.

Specifically, they might elucidate the enabling conditions for the formation of dynamic or stationary systems, manifested by the extent of ranking fortification by the top-ranked nodes. These results not only deepen our comprehension of complex systems’ popularity dynamics, but might also provide policy-makers and regulators with intervention means for tuning systems’ properties in order to control the extent of their internal mobility.

## 2. Results

In this study, we analyse and compare temporal aspects of nodes’ *relative* popularity levels (as viewed through their degree-based rankings) to the temporal dynamics of their *absolute* popularities. In order to investigate the dynamics of both popularity measures we first analyse their emergence through several empirical datasets (full details listed in §4.3):

1. Amazon products rankings;
2. Ethereum Blockchain trading ledger;
3. eToro trading platform.

In particular, we refer to each empirical dataset as an evolving network, consisting of weekly snapshot graphs for each time-stamp *t*, {*G*_*t*_ = (*V*_*t*_, *E*_*t*_)}_*t*∈[*T*]_ (a formal definition can be found in §4.1).

### 2.1. Absolute versus relative popularity dynamics

Consistently with previous works, we find clear preferential attachments and detachment patterns with respect to absolute popularity dynamics, manifested through nodes’ degrees, across all examined datasets. Specifically, we demonstrate that the average increase in a node’s *v* degree during the next time-stamp *t* + Δ*t* is a monotonic function of its current degree deg_*t*_(*v*), expressing preferential attachment principles. Formally, we denote by *D*_↑_(*d*) the average degree rise, across all time-stamps, of all nodes with current degree *d* whose degree will rise in the upcoming snapshot.

**Figure 1.**
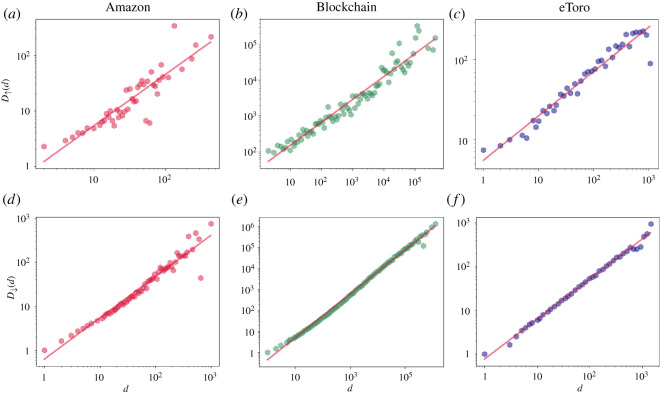
Average magnitude of degree change during the next time-stamp as a function of the previous degree, examined over three empirical datasets: Amazon (*a*,*d*), Blockchain (*b*,*e*) and eToro trading platform (*c*,*f*). Panels (*a*–*c*) depict the average magnitude of degree rise *D*_↑_ and (*d*–*f*) present average magnitude of degree drop *D*_↓_, as a function of the previous degree *d*. Across all panels, *d* values are binned logarithmically, where the markers represent the average *D*_↑_ (and average *D*_↓_, correspondingly) for each bin. The dynamics of the presented degree changes follow monotonic patterns across all examined datasets (indicated by the red regression line in all plots), affirming preferential attachment and detachment principles.

**Note:** While preferential attachment principles hold on the average case, they are not necessarily manifested at the single node level for two main reasons: (i) stochastic effects are at play and (ii) mixed effects of preferential attachment and detachment forces which apply at the single node level. For these reasons, even if node A had a larger degree than node B at time *t*, node A may result with a lower degree than node B at time *t* + Δ*t*.

Figure 1*a*–*c* manifests the monotonic dependency of *D*_↑_ on the current node degree *d*. Similarly, we affirm preferential detachment effects by presenting the monotonic dependency of the average degree drop, *D*_↓_(*d*), on the current node degree. Figure 1*d*–*f* depicts clear preferential detachment patterns, manifested by the monotonic dependency of *D*_↓_ on the current degree *d*.

**Figure 2.**
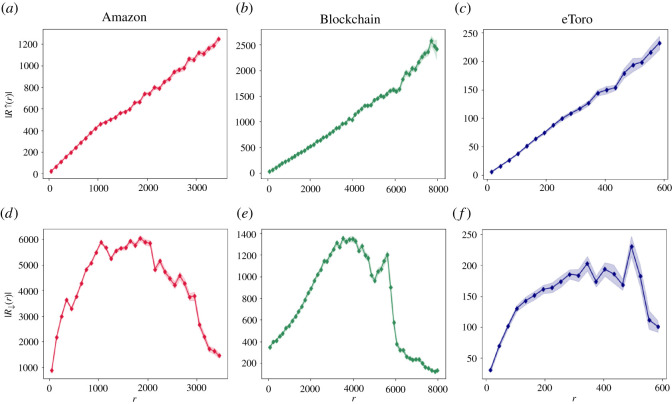
Average magnitude of rank change at the next time-stamp as a function of current rank, examined over three empirical datasets: Amazon (*a*,*d*), Blockchain (*b*,*e*) and eToro (*c*,*f*). Upper panels depict the average magnitude of rank improvement *R*_↑_ and lower panels depict the average magnitude of rank deterioration *R*_↓_ as a function of the current rank *r*. Across all panels, *r* values are linearly binned, where the markers represent the average *R*_↑_ (and correspondingly *R*_↓_) for each bin. The light background colour in each panel depicts ±1 standard error from the average binned values. The presented *R*_↑_ and *R*_↓_ dynamics across all examined datasets do not follow preferential attachment principles.

We set our first goal in examining whether relative popularity dynamics exhibit similar preferential attachment and detachment patterns. Specifically, for each snapshot network *G*_*t*_ we rank all nodes in *v* ∈ *V*_*t*_ according to their associated degree, in a descending order.^3^ We examine the average improvement in a node’s *v* rank during the next time-stamp *t* + Δ*t*, as a function of its current rank *rank*_*t*_(*v*). Formally, we denote by *R*_↑_ (*r*) the average rank improvement, across all time-stamps, for all nodes with current rank *r* whose rank will improve in the upcoming time-stamp. Figure 2*a*–*c* manifests a monotonic dependency of *R*_↑_ on the current rank *r*, across all examined datasets. While the improvement of both absolute and relative popularities present monotonic patterns, it is important to note that they are anti-correlated. Indeed, while better-ranked/higher-degree nodes present high improvement levels for the absolute popularity case, they exhibit low improvements for the relative case. Similarly, whereas worse-ranked/lower-degree nodes present low improvement levels for the absolute popularity case, they exhibit high improvements for the relative case.

We further analyse the dependency of the average rank deterioration *R*_↓_ (*r*) on the current node rank. Figure 2*d*–*f* depicts *R*_↓_ (*r*) as a function of its current rank, demonstrating non-monotonic dynamics, across all three empirical datasets. This indicates that unlike the exhibited monotonous absolute popularity dynamics, the deterioration in relative popularity does not follow preferential principles, but rather adheres to an inverse-U shaped curve.

### 2.2. Relative popularity: stability and volatility zones

After establishing the fundamental difference between the dynamics of these two highly related popularity measures, we turn to analyse the overall stability exhibited by nodes’ rankings. Specifically, for any given rank *r*, we examine the expected rank change during the next time-stamp *t* + Δ*t*, $R_{↕}$, as a function of the current rank *r*.

**Figure 3.**
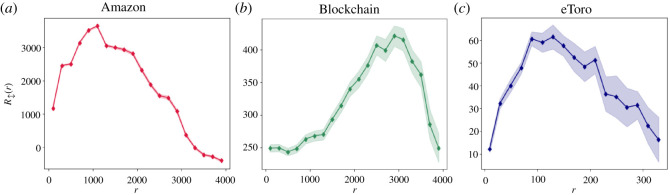
Average rank change at the next time-stamp as a function of current rank. Calculated on three empirical datasets. Panel (*a*) depicts the average rank change of the Amazon dataset, panel (*b*) presents Blockchain ranking changes, and panel (*c*) reflects the eToro dataset ranking changes. Across all panels, *r* values are linearly binned, where the markers represent the average $R_{↕}$ for each bin. The light background colour in each panel depicts ±1 s.e. from the average binned values. All three datasets follow inverse-U patterns.

We start by examining how the rank changes as a function of the current rank across all three empirical datasets.^4,^^5^ Figure 3 depicts a node’s average rank change depending on its current rank, demonstrating non-monotonic dynamics. This indicates that unlike the exhibited monotonous absolute popularity dynamics, relative popularity does not follow preferential principles, but rather adheres to an inverse-U shaped curve.

In order to substantiate our findings, we perform an analytical derivation of $R_{↕}$. To this end, we refer to the network as a system of competing agents *S*(*N*, *H*, *γ*), where *N* stands for the number of agents (nodes) in the system, *H* for the number of competitions performed (representing the number of edges added to the system in the next time-stamp), and *γ* for the power-law degree distribution parameter. A full analytical derivation of the expected rank change, based on this representation is presented in §4.2.

**Figure 4.**
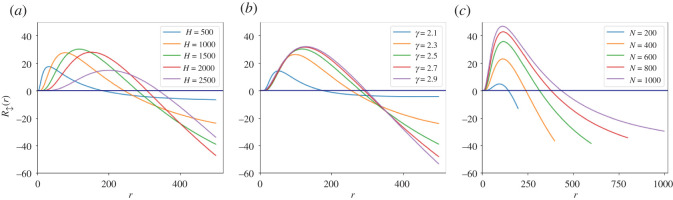
Expected rank change in the next time-stamp $R_{↕}$ as a function of the current rank *r*. Panel (*a*) presents model evaluations given fixed *γ* and *N* with various *H* values. Panel (*b*) is based on fixed *N* and *H* and various *γ* values. Panel (*c*) depicts evaluations based on fixed *H* and *γ* while *N* changes. All constellations manifest evident inverse-U patterns, each with different curve properties. At all times where constant values were used, we employed: *N* = 500, *H* = 1500, *γ* = 2.5.

Figure 4 presents evaluations of $R_{↕}$ analytical derivation as a function of the current rank, calculated using various parameter values for *H* (*a*), *γ* (*b*) and *N* (*c*). While the exact numerical characteristics of each plot vary with the chosen parameters, the entire range of examined parameters presents an inverse-U shaped curve. This finding is in complete consistency with our empirical analysis and substantiates our hypothesis regarding the non-monotonic dynamics of relative popularity of nodes, as opposed to those established by absolute popularity.

**Figure 5.**
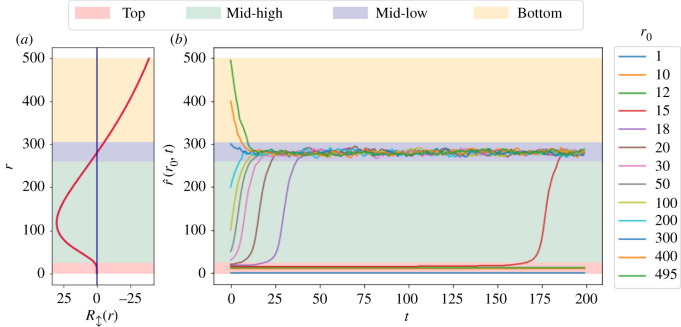
Node stability characteristics and prognosis. Panel (*a*) depicts the U-curved ranking evolution established by the agents-based system using the parameters: *γ* = 2.5, *H* = 1500, *N* = 500. It suggests four stability categories of node rankings: Top, Mid-high, Mid-low and Bottom ranked nodes. Panel (*b*) presents the expected rank of nodes, $\hat{r}(r_{0},t)$, as a function of various initial ranks (*r*_0_) and time (*t*). The exhibited dynamics ascertains the convergence of system nodes into one of two stability categories (Top and Mid-low ranked).

The exhibited inverse-U shaped ranking evolution suggests a qualitative structural partition of the node set, each characterized by different stability properties. Specifically, as depicted in figure 5*a*, the node set can be split into four categories, controlling the mobility potential of the nodes within, based on their current popularity:

1. *Top-ranked* nodes present high stability in terms of popularity and will probably experience only minor changes to their future popularity levels (red background).
2. *Mid-high ranked* nodes exhibit high instability, manifesting high expected drops to their future ranks (green background).
3. *Mid-low ranked* nodes are highly stable and are expected to preserve their rank in the future (purple background).
4. *Bottom-ranked* nodes also display high instability, with high expected rises to their future ranks (yellow background).

Finally, we examine the expected long-term changes in node ranking, by iteratively applying our agents-based model. Specifically, given an initial rank *r*_0_, we evaluate its expected rank after *t* timesteps by calculating

2.1 $$\hat{r}(r_{0},t)=\hat{r}(r_{0},t-1)+R_{↕}(\hat{r}(r_{0},t-1))+\mathrm{Noise}(R_{↕}(\hat{r}(r_{0},t-1))),$$

where

2.2 $$\hat{r}(r_{0},0)=r_{0},$$

and Noise(*x*) is some Gaussian noise with *μ* = *x* and *σ* = 0.01*x. Figure 5*b* presents the expected rank of nodes with various initial ranks, *r*_0_, over time. The established long-term dynamics suggests that system nodes are expected to converge into one of two stable popularity categories—Top ranked and Mid-low ranked. We deduce that these two categories function as focal attraction zones, impeding massive ranking changes to nodes after entering them. Furthermore, the latter implies that nodes that manage to ‘climb the ranking ladder’ and reach its top, are significantly more likely to fortify their ranks, despite the potential massive fluctuations in their absolute network degrees.

## 3. Discussion

In this work, we examined the temporal dynamics of nodes’ popularity levels, manifested both through their actual degrees and by their relative degree-based rankings. Surprisingly, we attested to a fundamental difference between these two strongly related measures. We first demonstrated how preferential attachment and detachment forces govern temporal changes in nodes’ degrees and give rise to a well-expected monotonic dynamics. We established, however, both empirically and analytically that nodes’ ranking dynamics is dominated by other forces, which result in a strictly non-monotonous inverse-U shaped dynamics.

To better comprehend the intuition behind the inverse-U patterns evident in the dynamics of ranking, we re-employ the agents-based system presented in definition 4.5. We first suggest an explanation to the positive derivative of $R_{↕}(r)$ around top-ranked nodes, i.e. for *r* ≪ *N*. Given two top-ranked agents ranked 1 and 2, we note that both are ‘playing the same game’. Namely, in order to win in the next time-stamp, each is required to obtain merely one edge more than its opponent. However, although the difference in ranks between the two agents is only 1, their degree delta may be significantly higher, implying they toss extremely different coins, representing their success probability at acquiring new edges. Specifically, this competition is very much biased towards the top-ranked agent, since its degree in the previous time-stamp was significantly higher than his predecessor’s (due to the underlying power-law degree distribution). This results in a negligible probability for both agents to switch their ranking during the next time-stamp, and correspondingly in a positive derivative of $R_{↕}(r)$ for *r* ≪ *N*. By contrast, while the bottom-ranked (ranked *N*) and second bottom-ranked agents (ranked *N* − 1) are also playing the same game, they have approximately the same coin. Namely, due to the underlying power-law distribution, both nodes are expected to have roughly the same degree in the previous time-stamp. Moreover, while the bottom-ranked agent can only maintain or improve its ranking during the next time-stamp, its peer has the potential to drop in rank. Therefore, the expected next rank of the *N*th ranked agent is better than that of the (*N* − 1)th, resulting in a negative derivative of $R_{↕}$ around *N*. Combining with calculus considerations,^6^ we conclude that there exists a rank $\bar{r}$ such that $1\leq \bar{r}\leq N$ on which $R_{↕}(\bar{r})$ is maximized.

Our agent-based model simulations also suggest that the expected change in rank and the boundaries of the stability categories strongly depend on the system’s characteristics: *N*, *H* and *γ*. Nevertheless, an analytical investigation of the dependency between the demonstrated inverse-U ranking evolution and the aforementioned system’s characteristics is still lacking. Specifically, deriving analytical approximations of the $R_{↕}$ expression and establishing upper and lower bounds to it, might support our empirical and experimental findings, substantiate the dependencies among system properties and indicate as to extreme cases in which the inverse-U dynamics does not formulate.

An additional essential research effort might be directed towards the comparison between empirical findings and the theoretical formulation. Since each system’s parameters *γ*, *N*, *H* can be easily extracted, these can be used to evaluate the expected inverse-U curve achieved by our agents-based model. In this work, we established preliminary results, demonstrating an excellent fit between the eToro empirical dataset and the *S*(*N*, *H*, *γ*) model (electronic supplementary material, section A, fig. 6). However, both Amazon and Blockchain datasets have extremely large parameters (*N*, *H*) and could not be fitted by our current implementation, due to computational issues. Reducing our model’s computational complexity or using it to fit other small datasets are prospective research leads.

Lastly, it is of great interest to verify how system stability levels (as apparent from the ranking change dynamics) are affected by time. Namely, whether a system presents different stability patterns when examined along different periods. In this study, we established preliminary results, while examining 1-year-long intervals of the Amazon dataset. Electronic supplementary material, section C, fig. 8 depicts $R_{↕}$ as established for six different year-long periods. Interestingly, the system follows an inverse-U shaped curve across all examined intervals; however, the characteristics of these curves change along time. Indeed, this initial temporal analysis opens an intriguing direction of future research, by examining system evolution, in terms of its ranking stability characteristics over time. The authors intend to pursue all above-stated directions in a future research.

Very few previous studies also investigated stability patterns in ranking evolution; however, they differ considerably from our current results. For instance, Ghoshal & Barabasi [14] demonstrated the existence of stability in node ranking after performing ‘deliberated’ network re-wiring. In this study, we focused on natural evolution dynamics of networks, without externally induced perturbations. Blumm *et al.* [15] explored the required conditions for the stability of top-ranked items (only) over time, while our study presents system-level results, demonstrating the stability characteristics of the *entire* node set.

Numerous studies over the years demonstrated monotonic patterns in network-structured systems. To the best of our knowledge, this study provides the first demonstration of the existence of a non-monotonic structural network dynamics. Surprisingly, we demonstrate analytically how this non-monotonic dynamics is a direct derivation of the well-established monotonic preferential attachment principles. This suggests that many researchers’ nearly axiomatic expectation for monotonous structural network patterns needs amendment.

Lastly, the analyses presented in this paper assumed network-based systems. Nevertheless, the potential impact of our findings transcends beyond mere networks, and is applicable to other systems which may lack an explicit network structure. Indeed, the prerequisites of the presented analyses are the existence of a preferential attachment mechanism, with absolute values distributed according to a power-law distribution. In principle, any system adhering to these two prerequisites is expected to display similar categorical stability patterns. An interesting future direction would be to investigate the dynamics of such systems and re-confirm our findings in such settings.

To conclude, our findings can help in better understanding ranking evolution and internal system mobility patterns. Specifically, it may provide a plausible explanation as to why superstar nodes are able to fortify their ranks, why star nodes are more likely to fall, and why in certain systems bottom-ranked nodes have a feasible chance of improving their ranks, whereas in others this is almost impossible. Beyond a mere better system comprehension, our findings may also provide policy-makers and regulators with intervention and control means that would assist in directing systems towards a more inclined dynamics.

## 4. Methods and data

### 4.1. Notations and definitions

We start by defining an evolving network {*G*_*t*_ = (*V*_*t*_, *E*_*t*_)}_*t*∈[*T*]_ as follows:

**Definition 4.1.**

A temporal graph *G*_*t*_(*V*_*t*_, *E*_*t*_) for a given time *t* and a time-delta Δ*t*, is the undirected graph constructed from all transactions performed during the time period [*t*, *t* + Δ*t*). The set of vertices *V*_*t*_ consists of all entities participating in the network activity during that period,

4.1 $$V_{t}\;:\;=\{v\;\|\;v\text{ participates in network activity during }[t,t+\Delta t)\},$$

and the set of edges *E*_*t*_⊆ *V*_*t*_ × *V*_*t*_ is defined as

4.2 $$E_{t}\;:\;=\{(u,v)\|\text{An activity between }u\text{ and }v\text{ occurred during }[t,t+\Delta t)\}.$$

The temporal degree of a vertex *v* ∈ *V*_*t*_ refers to the in-degree, standing for the number of a node’s incoming edges during a given time. Formally defined as

4.3 $$\deg_{t}(v)=|\{u\in V_{t}|(u,v)\in E_{t}\}|.$$

Next, we define the average magnitude of a node’s degree drop and rise as a function of the node’s previous degree.

We formally define:

**Definition 4.2.**

Let {*G*_*t*_ = (*V*_*t*_, *E*_*t*_)}_*t*∈[*T*]_ be an evolving network. Given d∈N, denote by $V_{t}^{d↓}$ the subset of nodes that obtained a degree *d* at time *t* and will drop in degree in *t* + Δ*t*:

4.4 $$V_{t}^{d↓}=\{v\in V_{t}\cap V_{t+\Delta t}\;\;|\deg_{t}(v)=d\wedge \deg_{t+\Delta t}(v)<d\}.$$

The average degree drop of nodes in $V_{t}^{d↓}$ across all time-stamps (i.e. the downward degree changes over all time-steps, averaged only for nodes decreasing in degree) is

4.5 $$D_{↓}(d)=\frac{\sum_{t\in [T]}\sum_{v\in V_{t}^{d↓}}(d-\deg_{t+\Delta t}(v))}{\sum_{t\in [T]}|V_{t}^{d↓}|}.$$

Analogically, denote by $V_{t}^{d↑}$ the subset of nodes that obtained temporal degree *d* and will rise in degree in *t* + Δ*t*

4.6 $$V_{t}^{d↑}=\{v\in V_{t}\cap V_{t+\Delta t}|\deg_{t}(v)=d\wedge \deg_{t+\Delta t}(v)>d\}.$$

The average degree rise of all nodes in $V_{t}^{d↑}$ (across all time-stamps) is

4.7 $$D_{↑}(d)=\frac{\sum_{t\in [T]}\sum_{v\in V_{t}^{d↑}}(\deg_{t+\Delta t}(v)-d)}{\sum_{t\in [T]}|V_{t}^{d↑}|}.$$

Similarly, we turn to define the average magnitude of rank change as a function of the previous rank.

**Definition 4.3.**

Given a time-stamp *t* ∈ [*T*], the rank assigned to node *v* ∈ *V*_*t*_, according to its degree deg_*t*_(*v*), is denoted by rank_*t*_(*v*). The ranking is performed in a descending order, such that the rank of 1 corresponds to the highest degree node in *V*_*t*_. Specifically, rank_*t*_(*v*) = *r* if there are *r* − 1 nodes with a higher degree than *v*

4.8 $${\mathrm{rank}}_{t}(v)=|\{u\in V_{t}\text{ such that }\deg_{t}(u)>\deg_{t}(v)\}|+1.$$

Ties are broken randomly, by a random internal ranking of groups containing identical degree nodes.

**Definition 4.4.**

Let {*G*_*t*_ = (*V*_*t*_, *E*_*t*_)}_*t*∈[*T*]_ be an evolving network. Given a rank r∈N and a time-stamp *t* ∈ [*T*], denote by $V_{t}^{r↓}$ the subset of nodes that obtained a rank *r* at time *t* and will deteriorate in rank at *t* + Δ*t*

4.9 $$V_{t}^{r↓}=\{v\in V_{t}\cap V_{t+\Delta t}|{\mathrm{rank}}_{t}(v)=r\wedge {\mathrm{rank}}_{t+\Delta t}(v)>r\}.$$

The average deterioration in rank *r*, after a Δ*t* time is denoted by

4.10 $$R_{↓}(r)=\frac{\sum_{t\in [T]}\sum_{v\in V_{t}^{r↓}}({\mathrm{rank}}_{t+\Delta t}(v)-r)}{\sum_{t\in [T]}|V_{t}^{r↓}|}.$$

Analogically, denote by $V_{t}^{r↑}$ the subset of nodes that obtained temporal rank *r* and will improve in rank at *t* + Δ*t*

4.11 $$V_{t}^{r↑}=\{v\in V_{t}\cap V_{t+\Delta t}|{\mathrm{rank}}_{t}(v)=r\wedge {\mathrm{rank}}_{t+\Delta t}(v)<r\}.$$

The average improvement in rank *r* after a Δ*t* time is denoted by

4.12 $$R_{↑}(r)=\frac{\sum_{t\in [T]}\sum_{v\in V_{t}^{r↑}}({\mathrm{rank}}_{t+\Delta t}(v)-r)}{\sum_{t\in [T]}|V_{t}^{r↑}|}.$$

Furthermore, we denote by $V_{t}^{r↕}$ the subset of nodes that obtained temporal rank *r* at time *t*

4.13 $$V_{t}^{r↕}=\{v\in V_{t}\cap V_{t+\Delta t}|{\mathrm{rank}}_{t}(v)=r\}.$$

The average change in rank *r*, after Δ*t* time is denoted by

4.14 $$R_{↕}(r)=\frac{\sum_{t\in [T]}\sum_{v\in V_{t}^{r↕}}({\mathrm{rank}}_{t+\Delta t}(v)-r)}{\sum_{t\in [T]}|V_{t}^{r↕}|}.$$

**Note:** Each temporal network has at most one node achieving any given rank, due to the random tie-breaking mechanism. As a result, $V_{t}^{r↑}$, $V_{t}^{r↓}$ and $V_{t}^{r↕}$ each contain either a single node, or none, as opposed to $V_{t}^{d↑}$ and $V_{t}^{d↓}$ (defined in definition 4.2).

For convenience, a pseudo-code for calculating both $rank_{t}(v),\forall \;t\in [T]$ and $R_{↕}(r)$ is available in the electronic supplementary material, section F.

Finally, we formulate the evolving network as a system of agents, defined as:

**Definition 4.5.**

Let {*G*_*t*_ = (*V*_*t*_, *E*_*t*_)}_*t*∈[*T*]_ be an evolving network. We denote by *S*_*t*_(*N*, *H*, *γ*) the representation of this evolving network as a system of competing agents at a given time-stamp *t*∈ [*T*]:

1. *S*_*t*_ consists of *N* potential agents, representing all vertices up to time-stamp *t*: $v\in \underset{\tau \leq t}{⋃}V_{\tau }$.
2. During [*t*, *t* + Δ*t*) the agents conduct *H* Bernoulli trials. A trial is considered successful if the corresponding agent was able to attract an edge. *H* = |*E*_*t*_|, the amount of edges added to the network during [*t*, *t* + Δ*t*).
3. Each agent’s probability of success depends on its associated node’s previous degree, and coincides with preferential attachment assumptions, based on a power-law degree distribution with a *γ* parameter.

### 4.2. Ranking change analysis

Considering an evolving network {*G*_*t*_ = (*V*_*t*_, *E*_*t*_)}_*t*∈[*T*]_, we derive the analytical form of $R_{↕}(r)$, the expected change in rank^7^ after a Δ*t* period of time, for a node of rank *r*, as a function of *r*. Given *t* ∈ [*T*], the rank change of $v_{t}^{r}$ clearly depends on the number of nodes that had a worse rank than $v_{t}^{r}$ in time-stamp *t* and a better rank than $v_{t}^{r}$ after Δ*t* time:

4.15 $$A_{t}^{r}=\{u\in V_{t}\cap V_{t+\Delta t}|{\mathrm{rank}}_{t}(u)>r>{\mathrm{rank}}_{t+\Delta t}(u)\},$$

and on the number of nodes that had a better rank than $v_{t}^{r}$ in time-stamp *t* and a worse rank than $v_{t}^{r}$ after Δ*t* time:

4.16 $$B_{t}^{r}=\{u\in V_{t}\cap V_{t+\Delta t}|{\mathrm{rank}}_{t}(u)<r<{\mathrm{rank}}_{t+\Delta t}(u)\}.$$

We correspondingly define the following indicator random variables:

4.17 $$1_{\left\{A_{t}^{r}\right\}}(v)=\left\{ \begin{array}{cc} 1\; & \text{if}\;v\in A_{t}^{r}\;\\ 0\; & \text{otherwise}\; \end{array}\right.,$$

and

4.18 $$1_{\left\{B_{t}^{r}\right\}}(v)=\left\{ \begin{array}{cc} 1\; & \text{if}\;v\in B_{t}^{r}\;\\ 0\; & \text{otherwise}\; \end{array}\right.$$

Incorporating the above we get

4.19 $$R_{↕}(r)=\underset{t\in [T]}{E}[R_{t+\Delta t}(v)-r]$$

4.20 $$=\underset{t\in [T]}{E}[1_{\left\{A_{t}^{r}\right\}}]-\underset{t\in [T]}{E}[1_{\left\{B_{t}^{r}\right\}}]$$

4.21 $$=\sum_{\;j=r+1}^{N}P_{\mathrm{switch}}(r,j)-\sum_{\;j=1}^{r-1}P_{\mathrm{switch}}(j,r),$$

where *P*_switch_(*j*, *k*) stands for the probability that two vertices ranked *j* and *k* switched their order of ranking after Δ*t* time.

In order to estimate *P*_switch_ we turn to the agents-based representation of the evolving network, introduced in definition 4.5. Specifically, we consider a system consisting of *N* nodes^8^ at a given time *t* where *H* edges are to be added along the following timespan Δ*t*, resulting in |*E*_*t*_| = *H*. Each agent represents such potential node which performs *H* Bernoulli trials during Δ*t*, where a successful experiment stands for acquiring an edge. The agent’s success probability at *t* + Δ*t* strictly depends on the temporal degree of its associated node at time *t*, in compliance with the system’s degree preferential attachment assumption

4.22 $$p_{t+\Delta t}(v)=\frac{\deg_{t}(v)}{\sum_{u\in V_{t}}\deg_{t}(u)}.$$

The probability of a node *v* ∈ *V*_*t*+Δ*t*_ to attract *k* out of a total of *H* edges, with a success probability *p*_*t*+Δ*t*_(*v*) can be modelled by a Binomial distribution. Let $Bin_{v}^{t+\Delta t}$ denote such random Binomial variable with parameters *H*, *k* and *p*_*t*+Δ*t*_(*v*):

4.23 $$\mathrm{Pr}({\mathrm{Bin}}_{v}^{t+\Delta t}=k)=\left(\begin{array}{c} H\\ k \end{array}\right)(p_{t+\Delta t}{\left(v)\right)}^{k}{\left(1-p_{t+\Delta t}(v)\right)}^{H-k}.$$

For clarity and simplicity, given a node *v* ∈ *V*_*t*+Δ*t*_ ranked *j* we will use the following abbreviated notation:

$$p_{j}=p_{t+\Delta t}(v),\;{\mathrm{Bin}}_{j}^{t+\Delta t}={\mathrm{Bin}}_{v}^{t+\Delta t}.$$

We note that for a node ranked *k* to outrank node ranked *j*, the difference between the success amounts of their corresponding agents should be strictly positive

4.24 $${\mathrm{Bin}}_{k}^{t+\Delta t}-{\mathrm{Bin}}_{j}^{t+\Delta t}>0.$$

Subsequently, for two nodes *u*, *v* ∈ *V*_*t*+Δ*t*_ ranked *j* and *k* correspondingly, where *j* < *k* we get

4.25 $$\begin{array}{rl} P_{\mathrm{switch}}(j,k) & =\mathrm{Pr}[{\mathrm{Bin}}_{j}^{t+\Delta t}<{\mathrm{Bin}}_{k}^{t+\Delta t}]\\ & =\sum_{l=1}^{H}\mathrm{Pr}[{\mathrm{Bin}}_{k}^{t+\Delta t}-{\mathrm{Bin}}_{j}^{t+\Delta t}=l]\\ & =\sum_{l=1}^{H}\sum_{i=0}^{(H-l)/2}\mathrm{Pr}({\mathrm{Bin}}_{j}^{t+\Delta t}=i)\times \mathrm{Pr}({\mathrm{Bin}}_{k}^{t+\Delta t}=l+i)\\ & =\sum_{l=1}^{H}\sum_{i=0}^{(H-l)/2}\left(\begin{array}{c} H\\ i \end{array}\right)p_{j}^{i}{\left(1-p_{j}\right)}^{H-i}\times \left(\begin{array}{c} H-i\\ l+i \end{array}\right)p_{k}^{l+i}{\left(1-p_{k}\right)}^{H-2i-l}. \end{array}$$

Combining (4.21) and (4.25) we receive

4.26 $$\begin{array}{rl} R_{↕}(r) & =\sum_{\;j=r+1}^{N}\sum_{l=1}^{H}\sum_{i=0}^{\frac{H-l}{2}}\left(\begin{array}{c} H\\ i \end{array}\right)p_{r}^{i}\times {\left(1-p_{r}\right)}^{H-i}\\ & \;\times \left(\begin{array}{c} H-i\\ l+i \end{array}\right)\times p_{j}^{l+i}\times {\left(1-p_{j}\right)}^{H-l-2i}\\ & \;-\sum_{\;j=1}^{r-1}\sum_{l=1}^{H}\sum_{i=0}^{\frac{H-l}{2}}\left(\begin{array}{c} H\\ i \end{array}\right)\times p_{j}^{i}\times {\left(1-p_{j}\right)}^{H-i}\\ & \;\times \left(\begin{array}{c} H-i\\ l+i \end{array}\right)p_{r}^{l+i}\times {\left(1-p_{r}\right)}^{H-l-2i}. \end{array}$$

In order to simplify the calculations, we have used an approximation of *p*_*j*_ for a node ranked *j* according to [14]

4.27 $$p_{j}=\frac{B(N-j+1,j-1/(\gamma -1))}{B(N-j+1,j)\cdot \hat{B}},$$

where $B(a,b)=\int_{0}^{1}y^{a-1}{\left(1-y\right)}^{b-1}\;dy$ is the Legendre beta function, *γ* is the power-law parameter of the degree distribution, *N* is the total number of nodes in the network at time *t* and $\hat{B}=\sum_{i=1}^{N}((B(N-i+1,i-(1/(\gamma -1))))/B(N-i+1,i))$ is the normalization factor.

### 4.3. Data

In this study, we validate our hypotheses regarding the dynamics of nodes’ absolute and relative popularity levels using various empirical datasets. In particular, we explore both degree and degree-based ranking changes of nodes, over the following empirical networks:

1. The *Amazon* rankings dataset [17]: this dataset contains product reviews and metadata from Amazon, including 142.8 million reviews spanning from May 1996 to July 2014. Previous studies that used this dataset for the modelling of network properties can be found for example in [18–21] We construct weekly bipartite networks, where nodes represent users and products, and edges represent individual ratings.
2. The *Blockchain* trading ledger: launched in July 2015 [22], the Ethereum Blockchain is a public ledger that keeps records of all Ethereum-related transactions. The ability of the Ethereum Blockchain to store not only ownership, similarly to Bitcoin, but also execution code, in the form of ‘Smart Contracts’, has led to the creation of a large number of new types of cryptocurrencies, based on the Ethereum ERC20 protocol. As a result, the ERC20 ecosystem constitutes one of the most fascinating examples for highly varied financial ecosystems, whose entire monetary activity is publicly available from its inception. This dataset has been previously demonstrated as a valuable information source for an abundance of network-theory-related studies [23,24] including financial assets adoption [25], malware and BOTs detection [26], and social signalling [27]. We have retrieved all transactions spanning from February 2016 to June 2018, resulting in over 88 million cryptocurrency trades, performed by over 17 million traders, trading more than 51 thousand different cryptocurrencies. We construct weekly bipartite networks containing cryptocurrency trading from the Ethereum Blockchain, where nodes represent traders and cryptocurrencies, and edges represent individual trades.
3. The *eToro* social trading platform^9^: an online social financial trading platform, allowing users to trade in currency, commodities and indices by selectively copying trading activities of prominent traders. This trading platform allows traders to take both long and short positions, with a minimal bid of as low as a few dollars, thus providing access for retail traders to investment activities, which until recently were only available for professional investors. Previous studies of this dataset can be found in [28–35]. The data that were analysed for this work encompassed approximately 3 million registered accounts, containing over 40 million trades during a period of 3 years. We build weekly networks in which nodes represent traders (followers and followees) and edges represent the copying action.

## Data Availability

Our paper uses data that were previously published elsewhere. All datasets are included in the electronic supplementary material [36].

## Footnotes

1 https://www.forbes.com/billionaires/; https://fortune.com/fortune500/.

2 Along this manuscript, we use the terms ‘preferential attachment’ and ‘rich get richer’ as an abuse of notation when applying them to the ranking of nodes. Specifically, due to the lower-bounded nature of ranks, a node cannot improve its rank beyond the first place, making it impossible for it to ‘get richer’. Here, we consider a generalized notion of these terms, referring to nodes that either improve or maintain their rank as those who become richer.

3 In this analysis, we break ties between identical degree nodes randomly. For an alternative tie-handling approach, the reader is referred to the electronic supplementary material, section B.

4 For establishing a clearer image of the inverse-U patterns, we present here a partial range only of *r* values. Consider the electronic supplementary material, section B for a complete range analysis.

5 Examining relative popularity dynamics of networks resulting from the Barabasi–Albert evolution model yields similar characteristics. Consider the electronic supplementary material, section C for further details.

6 https://en.wikipedia.org/wiki/Extreme_value_theory.

7 For the purpose of simplicity, we consider the expected rank change rather than the average change.

8 Not all *N* nodes will necessarily receive edges, resulting in |*V*_*t*+Δ*t*_| ≤ *N*.

9 https://www.etoro.com/.
